# GABAergic Neuron Specification in the Spinal Cord, the Cerebellum, and the Cochlear Nucleus

**DOI:** 10.1155/2012/921732

**Published:** 2012-06-28

**Authors:** Kei Hori, Mikio Hoshino

**Affiliations:** Department of Biochemistry and Cellular Biology, National Institute of Neuroscience, National Center of Neurology and Psychiatry (NCNP), 4-1-1 Ogahahigashi, Kodaira, Tokyo 187-8502, Japan

## Abstract

In the nervous system, there are a wide variety of neuronal cell types that have morphologically, physiologically, and histochemically different characteristics. These various types of neurons can be classified into two groups: excitatory and inhibitory neurons. The elaborate balance of the activities of the two types is very important to elicit higher brain function, because its imbalance may cause neurological disorders, such as epilepsy and hyperalgesia. In the central nervous system, inhibitory neurons are mainly represented by GABAergic ones with some exceptions such as glycinergic. Although the machinery to specify GABAergic neurons was first studied in the telencephalon, identification of key molecules, such as pancreatic transcription factor 1a (Ptf1a), as well as recently developed genetic lineage-tracing methods led to the better understanding of GABAergic specification in other brain regions, such as the spinal cord, the cerebellum, and the cochlear nucleus.

## 1. Introduction

The mammalian brain is a complex, highly organized structure that has a wide variety of morphologically and physiologically different neuronal cell types and diverse types of glia. Higher brain function is primarily accomplished by assembly of neural circuits with specific patterns of synaptic connectivity between diverse neuronal cell types. This fundamental process begins with cell fate determination, whereby progenitor cells in the ventricular zone exit the cell cycle and differentiate into distinct cell types with specific neuronal identities, followed by migration of the neuronal cells to proper regions in the brain, and axon guidance that extends to and recognizes their targets. There are two broad types of neurons, excitatory neurons and inhibitory neurons. In the central nervous system, excitatory neurons are mainly glutamatergic neurons that transmit information between different regions in the brain whereas inhibitory neurons are mainly composed of GABAergic and glycinergic neurons, make local connections, and are thought to act as a cellular elements coordinating and balancing excitatory activity. Indeed, previous studies have revealed that severe impairment of the GABAergic inhibitory system caused by deficits of genes regulating the development of GABAergic interneurons (e.g., transcription factor ARX [1] and Nkx2-1 [2]) or function of GABAergic neurotransmission (e.g., channels and transporters [3]) leads to pathological hyperexcitability and can result in severe epilepsy [4]. In other CNS regions, other types of neurotransmitters, such as histamine and taurine released from the hypothalamic inhibitory interneurons, also exert inhibitory actions [5–7]. Furthermore, shift of the neurotransmitter phenotype from GABAergic predominance to mainly glycinergic (or coreleased from single synaptic terminals) neurotransmission occurs in some neurons, such as the interneurons projecting onto spinal motoneurons and lateral superior olive auditory relay neurons in the brainstem during postnatal maturation of inhibitory system [8–10]. The impairment of the maturation of GABAergic neurotransmission to motoneurons in the spinal cord and brainstem is thought to induce neurological dysfunctions such as hyperekplexia and amyotrophic lateral sclerosis [8]. In addition to the principal inhibitory role of GABA in mature neurons in adults, the excitatory effects of GABA in immature neurons have been demonstrated in a broad range of CNS regions [8, 9, 11, 12]. In early stages of brain development, GABA exhibits depolarizing actions due to the efflux of chloride ions mediated through GABA_A_ receptors in immature neurons, due to a relatively higher intracellular versus extracellular concentration of chloride ions. The GABA-dependent neuronal excitation in the developing brain plays a fundamental role for trophic factors by influencing multiple developmental processes including neurite outgrowth, cell migration, and cell survival as well as instructive actions for the construction of neuronal circuits in the CNS [11, 13–17]. Taken together, dysfunction of GABAergic neurons such as the imbalanced generation of glutamatergic and GABAergic neurons is implicated as a cause of various neurodevelopmental disorders including epilepsy, hyperalgesia, and allodynia as well as seizures of the immature brain [11, 18–22].

The choice between the excitatory and inhibitory cell fates of progenitor cells is made by tightly controlled genetic programs. Elucidation of the mechanisms that control specific neuronal cell fate is fundamental for understanding how the central nervous system functions. Over the last decade, considerable progress has been made in defining the molecular mechanisms that control the balance of excitatory and inhibitory neuronal cell fate through recently developed mouse genetic lineage-tracing methods in addition to gene-transfer technologies. Progress in defining mechanisms underlying the development of cortical GABAergic interneurons has been well summarized in several recent reviews [23–25].

In this paper, we focus on the molecular mechanisms specifying GABAergic neuronal cell fate in the caudal part of CNS regions including the spinal cord, the cerebellum, and the cochlear nucleus, particularly from the viewpoint of transcriptional networks regulated by the homeodomain-type and proneural basic helix-loop-helix- (bHLH-) type transcription factors.

## 2. Specification of GABAergic Interneurons in the Dorsal Spinal Cord

 In the dorsal spinal cord, association and relay neurons in the dorsal spinal cord play essential roles in integrating incoming sensory information, including pain, temperature, and mechanoception, and transducing these into signals for motoneurons or higher brain centers. Functionally, GABAergic neurons in the dorsal spinal cord are involved in modulating the strength of sensory input to the spinal cord by presynaptic inhibition of primary sensory afferents [26]. 

**Figure 1:**
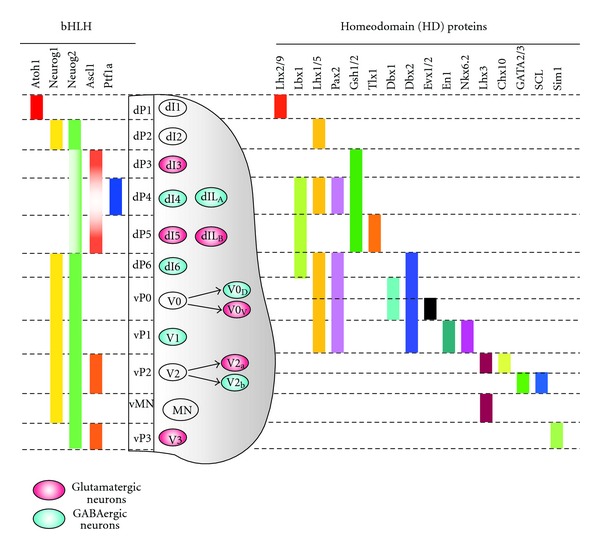
The specification of the spinal cord neurons directed by a combinatorial code of transcription factors. Schematic summary of the expression of bHLH transcription factors in the progenitor cells (left) and homeodomain (HD) transcription factors in the differentiating and differentiated neurons. Eleven early classes of postmitotic neurons (dI1~6, V0~3, and motoneurons (MN)) and two late-born dorsal interneurons (dIL_A_ and dIL_B_) are present in the embryonic spinal cord. The immature postmitotic V0 and V2 interneurons are further subdivided into two distinct interneuron subtypes indicated by arrows. The glutamatergic excitatory neurons and GABAergic inhibitory neurons are represented by red and blue circles, respectively.

 In the early developing neural tube during embryonic days 10–11.5 (E10–11.5), six distinct classes of deep dorsal interneurons (dI1–6) arise from six different progenitor domains (dP1–6), followed by the generation of two late-born neuronal subtypes of superficial laminae, dIL_A_ and dIL_B_, from a common dorsal progenitor domain during E11–13 (Figure 1) [27, 28]. While postmitotic dorsal interneurons can be distinguished by the characteristic combinatorial expression of homeodomain (HD) transcription factors like ventral interneurons described below [29, 30], proneural bHLH transcription factors have a predominant role for establishing their progenitor domains with an almost complementary and nonoverlapping pattern in the spinal cord [27, 28, 31, 32].

 GABAergic neurons in the dorsal spinal cord are composed of early-born dI4 and dI6 and late-born dIL_A_ neurons. These three classes of postmitotic interneurons express the HD transcription factor Lbx1, Pax2 and Lhx1/5 [29, 30]. A bHLH transcription factor Ptf1a plays a central role in the specification of these GABAergic inhibitory dorsal interneurons while suppressing the generation of excitatory glutamatergic interneurons [33, 34]. Mice lacking *Ptf1a* show a near complete loss of dI4 and dIL_A_ GABAergic interneurons while containing increased numbers of excitatory dI5 and dIL_B_ interneurons. In contrast, Ptf1a suppresses the HD factor Tlx3, which is an important postmitotic determinant for dorsal glutamatergic interneurons [35, 36]. Overexpression of Ptf1a in the chick neural tube can induce ectopic Pax2-positive inhibitory neurons at the expense of Tlx3-positive glutamatergic excitatory neurons dI5 and dIL_B_ [33, 34]. Another bHLH transcription factor, Ascl1, also participates in the specification of these neurons in a more complex way. Ascl1 and the HD factors Gsh1 and Gsh2 coordinately activate Tlx3 expression to promote the generation of dI5 glutamatergic interneurons in early developmental stages [37, 38]. In the late-born dIL populations, however, Ascl1 functions to antagonize Gsh1 and Gsh2 by upregulating Ptf1a expression and thus is necessary for the specification of dIL_A_ GABAergic interneurons in the dorsal horn [38, 39]. Furthermore, Ascl1 simultaneously activates Notch signaling in non-cell-autonomous manner that promotes dIL_B_ glutamatergic cell fate over a dIL_A_ cell fate. Pax2 is also an essential regulator for the differentiation of GABAergic inhibitory neurons, as demonstrated by *Pax2*-mutant mice in which GABAergic markers in the dorsal horn are drastically reduced [35]. Although Lhx1 and Lhx5 are not required for the initial specification of GABAergic neurons, these factors maintain Pax2 expression as well as inhibitory-neurotransmitter expression of genes such as Gad1 at later developmental stages [40]. Although Pax2 and Lhx1/5 seem to function downstream of Ptf1a, it remains to be determined whether these factors are direct or indirect downstream targets of Ptf1a. Recent studies have demonstrated that another bHLH transcription factor Neurog2 is a direct downstream target for Ptf1a [41].

## 3. Specification of GABAergic Interneurons in the Ventral Spinal Cord

 In the ventral spinal cord, motor neurons and several types of interneurons assemble into local networks that contribute to the generation of the rhythmic output required for locomotion [42]. In the early development of the neural tube, an extrinsic factor Shh is released from the notochord and floor plate that induces the patterning of five distinct ventral progenitor domains (p0, p1, p2, pMN, and p3) in a concentration-dependent manner [43]. Four cardinal classes of interneurons (V0, V1, V2, and V3) and motor neurons are produced from each progenitor domain that can be distinguished by combinatorial transcription factor expression (Figure 1) [43–45]. 

 Motor neurons are basically cholinergic [46]. Excitatory glutamatergic interneurons include V3 and a subset of the V2 and V0 interneuron population whereas inhibitory interneurons (GABAergic and glycinergic neurons) are generated from p0, p1, and p2 progenitor domains [42, 47].

While V0 populations, defined by the expression of Evx1/2, are commissural interneurons that extend axons contralaterally and rostrally for 2–4 spinal cord segments, V1 interneurons, marked by Engrailed-1 (En1) and Foxd3 expression, are inhibitory neurons that project axons ipsilaterally and rostrally [48–53]. The V1 interneurons are initially generated as a homogeneous GABAergic interneuron population in developing neural tubes [51] but subsequently differentiate into a range of inhibitory interneuron cell types, including Renshaw cells (RCs) and putative reciprocal Ia inhibitory interneurons [52, 54, 55].

 V0 interneuron populations constitute heterogeneous neurotransmitter phenotypes. A majority of V0 interneurons derived from the ventral half of the p0 domain (described as V0_D_ interneurons) express vesicular inhibitory amino acid transporter (VIAAT) and represent both GABAergic and glycinergic neurons whereas one-third of V0 interneurons derived from the dorsal p0 progenitor domain (V0_V_ interneurons) show an excitatory neuronal phenotype that expresses VGLUT2, a marker of glutamatergic interneurons [49, 50, 56]. They are defined by the absence or presence of the HD factor Evx1 expression, respectively [56]. 

 Progenitor cells of both V0 and V1 share a combinatorial expression of Pax6 and Dbx2 [57]. Dbx1 is, however, uniquely expressed in V0 progenitor cells and is considered an essential factor for the specification of V0 interneurons [50]. In mice lacking *Dbx1*, V0 interneurons are lost, and concomitantly V0_D_ and V0_V_ interneurons are respecified into Lbx1-positive dI6 dorsal interneurons and En1-positive V1 interneurons, respectively.

 In contrast, Nkx6.2 is expressed in V1 progenitor cells and is required for the specification of V1 neuronal fate, also it represses the generation of V0 interneurons [58]. *Nkx6.2*-mutant mice display an expansion of the Dbx1 domain into the ventral region where it is destined to be the V1 progenitor domain, followed by the loss of V1 interneurons and a concomitant increase of V0 interneurons. Therefore, Dbx1 and Nkx6.2 play an important role as molecular switches defining the progenitor domains for V0 and V1 interneuron subtypes.

 V2 interneurons generated from a homogenous p2 progenitor domain are subdivided into two distinct subtype interneurons: excitatory V2a glutamatergic interneurons, marked by the expression of HD factors Lhx3 and Chx10, and inhibitory V2b GABAergic interneurons, which are characterized by the expression of GATA2/3 and a bHLH transcription factor SCL [59–65]. The asymmetry of V2a versus V2b interneuron fate is initiated by Notch-Delta signaling in immature postmitotic V2 progenitors [63, 66, 67]. Notch receptor ligand Dll4-expressing progenitors give rise to V2a interneurons, maintaining Lhx3 expression while repressing GATA2. These cells simultaneously activate the transcriptional pathways downstream of Notch signaling for the specification of V2b interneuron fate of Notch-expressing progenitors. Forkhead transcription factor Foxn4 acts as a key regulator of V2b interneuron specification [66, 68]. *Foxn4*-mutant mice show loss of Dll4 expression and subsequent cell fate change from V2b to V2a. As downstream factors of Notch signal, both GATA2 and SCL consolidate the transcription pathways to acquire V2b subtype identity [60, 62, 69]. Forced expression of GATA2 in the chick neural tube induces ectopic formation of V2b interneurons while suppressing the generation of other neurons including V2a interneurons. Mice lacking *SCL* exhibit downregulation of GATA2 and deficiency in V2b interneurons, accompanied by overproduction of V2a interneurons. LIM-only protein LMO4 functions as a nucleation factor by assembling a LIM complex with GATA2, SCL, and cofactor NLI, and this transcriptional complex promotes the GABAergic V2b interneuron identity [70].

## 4. GABAergic Neuron Specification in the Cerebellum

 There are three major regions in the cerebellum: cortex, white matter, and nuclei. The cerebellar cortex includes several types of glutamatergic excitatory and GABAergic inhibitory neurons. Glutamatergic neurons are comprised of granule cells and unipolar brush cells (UBCs), while the GABAergic population includes Purkinje, Golgi, Lugaro, stellate, basket, and candelabrum cells. Cerebellar nuclei (CN) are comprised of three major types of neurons: large glutamatergic projection neurons (CN-Glu neurons), midsized GABAergic inhibitory projection neurons (CN-GABA-ION neurons), and small GABAergic interneurons (CN-GABA interneurons). CN-GABA-ION neurons extend their axons to the inferior olivary nucleus (ION) [71], while CN-Glu neurons send their axons to nuclei outside the cerebellum, including the red nucleus and the thalamus. These neurons mutually regulate each other's activity to achieve proper cerebellar function.

 During development, the neuroepithelium of the alar plate of rhombomere 1 (r1) generates all types of cerebellar neurons [72–75]. The dorsal-most part of the neuroepithelium, the roof plate, of r1 does not generate neurons but produces cells of the choroid plexus [76]. Cerebellar neuron-producing neuroepithelium can be divided into two regions: the rhombic lip (RL) and the ventricular zone (VZ). These two regions can be morphologically discriminated by a notch located on their border.

 In 1997, Ben-Arie et al. reported that a bHLH type transcription factor, Atoh1, is expressed in the rhombic lip and involved in cerebellar granule cell generation [77]. In contrast, our Cre-loxP recombination-based lineage tracing studies revealed that another bHLH type transcription factor, Ptf1a, is expressed in the cerebellar VZ, which produces most of the cerebellar GABAergic neurons including Purkinje, Golgi, basket, stellate cells, CN-GABA-ION neurons, and CN-GABA interneurons [78]. Ptf1a is required for GABAergic neuron production, as GABAergic neurons were not generated in *cerebelless*, Ptf1a loss-of-function mutants as well as Ptf1a-knockout mice. Furthermore, ectopic expression of Ptf1a by means of in utero electroporation caused ectopic production of GABAergic neurons from the dorsal telencephalic neuroepithelium. In addition, Pascual et al. reported that in the *Ptf1a*-null mutants, the fate of neurons produced from the VZ is changed to that of granule cells [79]. Moreover, a recent genetic fate mapping study using *Ascl1* ^CreER^-knock-in mice showed that minor cerebellar GABAergic neurons, such as Lugaro and candelabrum cells, are also derived from the cerebellar VZ [80]. These observations suggested that Ptf1a, expressed in the cerebellar VZ, determines GABAergic neuronal fate in the cerebellum. *PTF1A *was also identified as a causative gene for a human disease that exhibits permanent neonatal diabetes mellitus and cerebellar agenesis [81].

 On the other hand, Fishell's and Zoghbi's groups reported a molecular fate map of the derivatives of *Atoh1*-expressing neuroepithelial cells in the cerebellar RL [82, 83]. They showed that not only granule cells but also some CN neurons are derived from the RL, although they did not discriminate between neuron types in the CN. In their studies, development of RL-derived CN neurons was shown to be disrupted in the *Atoh1 *mutants. As GABAergic but not glutamatergic CN neurons were found to be derived from Ptf1a-expressing neuroepithelial cells in the VZ [78], this suggests that cerebellar glutamatergic neurons such as granule cells and CN-Glu neurons are derived from the RL. Accordingly, unipolar brush cells, which are glutamatergic, were also shown to emerge from the RL [84].

 Together, these studies indicate the presence of two molecularly defined neuroepithelial areas in the cerebellum, the Atoh1-expressing RL and the Ptf1a-expressing VZ, which generate glutamatergic and GABAergic neurons, respectively. Each bHLH transcription factor is involved in specifying the corresponding neuronal subtype in the cerebellum [85]. 

 Although some clarification of the machinery governing GABAergic neuronal subtype specification by Ptf1a has been provided, molecular mechanisms to specify each GABAergic subtype (e.g., Purkinje, Golgi, basket, stellate cells and CN-ION, CN-interneurons) remain unclear. Birthdating studies using ^3^H-thymidine and BrdU [86–90] as well as adenovirus [91] have revealed that each type of neuron is generated at distinct developmental stages. 

 With regard to GABAergic neurons, Purkinje cells are produced early (E10.5~13.5 in mice), Golgi cells a little later (E13.5~postnatal day P(0) in mice), and stellate/basket cells mainly perinatally [86–91]. The newest study by Sudarov et al. revealed that candelabrum cells are generated around P0, while GABAergic CN neurons arise at early stages (E10.5~11.5) [80]. In addition, somatic recombination-based clonal analyses suggested that Purkinje, Golgi, and basket/stellate cells as well as some CN neurons (probably GABAergic) belong to the same lineage [92, 93]. These data indicate that some temporal information in the neuroepithelium may be involved in specification of neuronal types in the VZ. However, the underlying molecular mechanisms have not yet been clarified.

**Figure 2:**
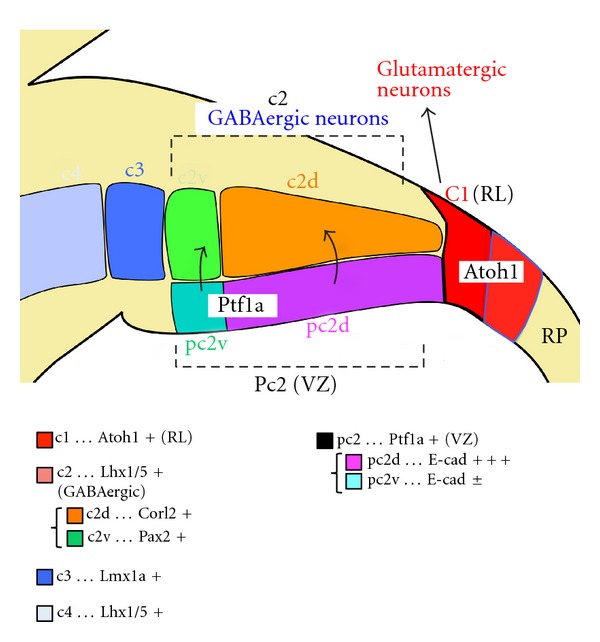
Domain structure of the cerebellar primordium. The c1 domain, expressing Atoh1, corresponds to the rhombic lip that produces all types of glutamatergic neurons in the cerebellum. The pc2 is the Ptf1a-expressing neuroepithelial domain that generates all types of GABAergic cerebellar neurons. At early neurogenesis stages, such as E12.5, the pc2 domain can be subdivided into pc2d and pc2v subdomains, which expresses E-cadherin strongly and weakly, respectively. The c2 domain, expressing Lhx1/5, consists of immature GABAergic neurons putatively generated from pc2 neuroepithelial domain. This domain can also be subdivided into two subdomains, c2d and c2v, corresponding to pc2d and pc2v, respectively. The c2d subdomain consists of corl2-expressing neurons or Purkinje cells, whereas the c2v subdomain includes Pax2-positive cerebellar GABAergic interneurons. Although c3 and c4 domains are Lmx1a and Lhx1/5 positive, respectively,cell types that consist these domains are unknown. The roof plate (RP) is located most dorsally and plays prominent roles in organizing this cerebellar domain structure.

 Some scientists have attempted to divide the structure of the cerebellar primordium into several domains (Figure 2). Chizhikov et al. defined four cellular populations (denoted as c1–c4 domains) in the cerebellar primordium via the expression of a few transcription factors [76]. c1 corresponds to the Atoh1-expressing RL, and c2 is located just above the Ptf1a-expressing VZ (denoted as pc2), indicating that c2 cells mainly consist of GABAergic inhibitory neurons. Although c3 and c4 express Lmx1a and Lhx1/5, respectively, their neuronal subtypes are still unknown. This domain structure is disrupted when the roof plate is removed [76]. Furthermore, at the early neurogenesis stage (e.g., E12.5 in mice), Minaki et al. subdivided the c2 domain into dorsally (c2d) and ventrally (c2v) located subdomains that express corl2 (also called Skor2) and Pax2, respectively [94]. While corl2 is exclusively expressed in immature and mature Purkinje cells [94], Pax2 is expressed in GABAergic interneurons (e.g., Golgi, stellate, basket, and CN-GABA neurons) in the cerebellum [95, 96]. They also subdivided the Ptf1a-expressing neuroepithelial domain (pc2) into pc2d and pc2v, which strongly and weakly express E-cadherin, respectively. From the positions of the neuroepithelial and neuronal subdomains, they suggested that the pc2d neuroepithelial subdomain produces cells in the c2d domain, which give rise to Purkinje cells, while the pc2v subdomain generates cells in the c2v that become GABAergic interneurons [97]. As development proceeds, pc2d and pc2v subdomains contract and expand, respectively, and by E14.5 in mice, the Ptf1a-expressing pc2 domain comprises only the pc2v subdomain, which expresses E-cadherin weakly. This correlates with the fact that, at E14.5, Ptf1a-expressing neuroepithelium does not produce Purkinje cells but Pax2-positive interneurons [91, 95]. The expression of several other transcription factors in the cerebellar VZ during development has also been reported. For example, Zordan et al. described the expression patterns of proneural bHLH transcription factors, such as Ngn1, Ngn2, and Ascl1, in the cerebellar VZ [98]. It has also been reported that Pax2-positive neurons, but not Purkinje cells, are reduced in the* Ascl1*-null cerebellum [99], while Purkinje cells are reduced in *Ngn1*-null mice [100], suggesting that these bHLH transcription factors play distinct roles in cerebellar development.

 In addition, several transcription factors have been reported to participate in the development of specific types of cerebellar neurons. Double knockout of *Lhx1* and *Lhx5* as well as the targeted disruption of their cofactor *Ldb1* resulted in lack of Purkinje cell production in the cerebellum although Pax2-positive interneurons did not seem to be affected. Because Lhx1 and Lhx5 are expressed in postmitotic cells, this suggests that Lhx1, Lhx5, and Ldb1 are postmitotically involved in Purkinje cell specification [101]. It is recently suggested that corl2 is involved in Purkinje cell maturation from analyses of loss-of-function mutants of corl2 [102]. In addition, in the *cyclin D2 *KO mice, the progenitor pool of GABAergic interneurons is precociously exhausted and progenitor numbers are significantly reduced, leading to a remarkable decrease in the number of late-born interneurons, such as stellate cells [103, 104]. 

 Heterotopic and heterochronic transplantation studies have also provided important clues to understanding cerebellar development [71]. When tissues from embryonic and postnatal cerebella were mixed and transplanted to the fourth ventricle of an adult mouse, the postnatal-derived cells differentiated only into interneurons such as granule, basket, and stellate cells, but not projection neurons, such as Purkinje cells, whereas the embryonic-derived cells were capable of becoming all types of cerebellar neurons [105]. It has also been shown that dissociated cells taken from cerebellar primordium at early neurogenesis stages can differentiate into all major types of cerebellar neurons, while those from postnatal cerebellum differentiated only to Pax2-positive interneurons [106]. These findings suggest that the differentiation competence of cerebellar progenitors becomes restricted as development proceeds. However, the molecular mechanisms underlying this fate restriction process have not yet been clarified. Interestingly, Leto et al. suggested that Pax2-positive interneurons, such as Golgi, stellate, basket cells, and CN-GABA interneurons, are derived from the same progenitor pool [89]. Leto et al. also clarified that, after leaving the VZ, progenitors for GABAergic interneurons continue to proliferate in the prospective white matter during late embryonic and postnatal development [107]. Their grafting studies showed that terminal commitment does not occur while precursors are still proliferating but occur postmitotically according to host-specific information, suggesting an instructive cue provided by the microenvironment of the prospective white matter.

## 5. GABAergic Neuron Specification in the Cochlear Nucleus

Sounds received in the ear are transmitted via the auditory nerve to the cochlear nucleus (CoN) of the mammalian hindbrain, where the auditory information is properly processed and relayed to the brain. The CoN is a very complex cell assembly that can be divided into two subregions, the ventral and dorsal cochlear nuclei (VCoN and DCoN), which differ in structure and feature. The DCoN exhibits a laminar and cerebellum-like architecture that includes a granule cell system whereas the VCoN does not have a laminar structure. Because of its importance in sound perception, the CoN has been intensely studied from anatomical, physiological, and histochemical points of view [108–110]. 

 Histological observations have deduced that a portion of neurons generated from the dorsal hindbrain neuroepithelia migrate tangentially to give rise to CoN neurons [111, 112]. More directly, genetic fate mapping studies using transgenic mice confirmed that many CoN cells are derived from the dorsal region of the hindbrain neuroepithelia where the *Wnt1* promoter is active [113, 114]. As to the rostro-caudal axis, the origins of CoN neurons seem to differ between birds and mammals. Grafting studies revealed that bird CoN neurons are derived from a broader part of the hindbrain (r3~r8), [115–117], while mouse genetic studies have suggested a more rostral and narrower origin (r2~r5) [113].

 Very sophisticated genetic fate mapping studies were carried out by Farago et al. [113] using an FLP-FRT and Cre-loxP-based dual lineage tracing system. In addition to showing that CoN neurons are derived from r2~r5, they also revealed that neurons in the anterior part of the VCoN (aVCoN), the posterior part of the VCoN (pVCoN), and the DCoN generally tend to be generated from rostral (~r2, 3), middle (~r3, 4), and caudal (~r4, 5) parts of the CoN neuron-producing hindbrain (r2~5), respectively, with some overlap.

 The CoN contains a variety of neurons that have distinct features [108–110]. For example, the DCoN includes GABAergic neurons (e.g., Golgi and molecular layer (ML) stellate cells), glycinergic neurons (e.g., cartwheel and tuberculoventral cells), and glutamatergic neurons (e.g., granule, unipolar-brush, giant and fusiform cells). The VCoN consists of glutamatergic neurons (e.g., Octopus, globular-bushy, spherical-bushy, and T-stellate cells) and glycinergic neurons such as D-stellate cells.

**Figure 3:**
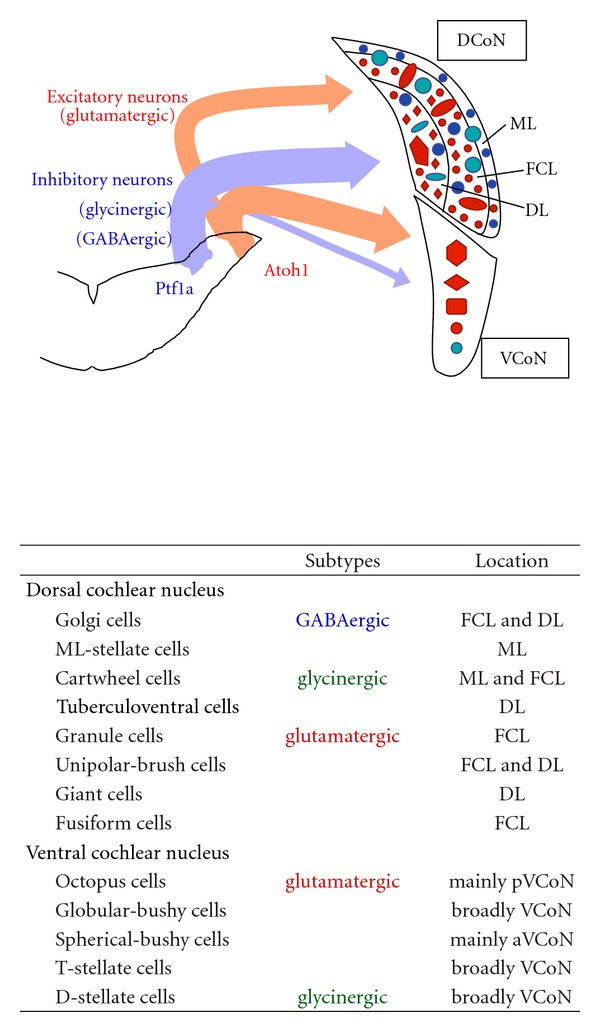
Lineages of excitatory and inhibitory neurons in the cochlear nucleus. (upper panel) Schematic of cochlear neuron lineages. Glutamatergic excitatory neurons are derived from the Atoh1-expressing RL whereas glycinergic/GABAergic inhibitory neurons are generated from Ptf1a-expressing neuroepithelial domain of the middle hindbrain (r2~5). (lower panel) Various cochlear nucleus neurons characterized by neurotransmitter subtype and location. DCoN: dorsal cochlear nucleus; VCoN: ventral cochlear nucleus; aVCoN: anterior VCoN; pVCoN: posterior VCoN; FCL: fusiform cell layer; ML: molecular layer; DL: deep layer.

 In the neuroepithelium of the middle hindbrain (r2~r5), Ptf1a and Atoh1 are expressed in distinct regions resembling the expression pattern in the cerebellum (Figure 3). Using Cre-LoxP-based genetic fate mapping studies, our group identified the origins of inhibitory and excitatory neurons of the cochlear nucleus; inhibitory (GABAergic and glycinergic) and excitatory (glutamatergic) neurons are derived from Ptf1a- and Atoh1-expressing neuroepithelial regions, respectively [118], and their development is dependent on the corresponding bHLH proteins. These findings suggest that Ptf1a and Atoh1 are involved in specifying inhibitory and excitatory neurons of the CoN, respectively, in a similar manner found in the cerebellum. However, little is known about the molecular machinery to generate distinct types of neurons with the same neurotransmitter, for example, Golgi and ML-stellate cells.

## 6. Conclusions and Future Perspectives

As described here, many recent studies have helped to clarify the molecular mechanisms controlling the specification of GABAergic neuronal cell fate in the hindbrain and spinal cord. While the patterning of the ventral spinal cord along the dorso-ventral axis is predominantly guided by combinatorial expression of HD transcription factors, in the hindbrain, including the cerebellum, the cochlear nucleus, and also the dorsal spinal cord, bHLH transcription factors play essential roles in not only patterning the progenitor domains but also specifying distinct neuronal subtypes. In the early developing dorsal spinal cord, distinct neuronal subtypes are defined by the specific expression of bHLH transcription factors including Atoh1, Neurog1/2, Ptf1a, and Ascl1 in their progenitor cells as well as the timing of their birth and different combinations of HD transcription factors. Among these factors, Ptf1a is a key molecule for the generation and specification of GABAergic interneurons among these factors. In the rostral (r1) and middle (r2~5) hindbrain, Ptf1a and Atoh1 are expressed in different neuroepithelial regions and participate in generating inhibitory and excitatory neurons, respectively. However, this rule is not applicable to the caudal (r6~r8) hindbrain. The Ptf1a neuroepithelial domain in the caudal hindbrain (r6~r8) produces not only inhibitory neurons (local circuit neurons) but also glutamatergic neurons (climbing fiber neurons) [119], while the Atoh1 domain generates glutamatergic mossy fiber neurons.

Despite the impressive progress in our understanding of the mechanisms controlling the balance of excitatory and inhibitory neuronal fate by these transcription factors in the hindbrain, many fundamental questions remain to be addressed. For example, although the requirement of Ptf1a for the appropriate balances of excitatory and inhibitory neurons in the hindbrain has been demonstrated, it remains unclear how Ptf1a diversifies the types of GABAergic inhibitory neurons generated from the common neuroepithelial regions during different developmental stages. Identification of downstream targets of Ptf1a will assist us in understanding the molecular mechanisms to specify each GABAergic neuronal subtype. In addition, we need to consider the regulation of bHLH function in other mechanisms such as posttranslational modification of transcription factors or epigenetic control of gene expression in the diversification of GABAergic neurons.
